# Factors that mediate and prevent degradation of the inactive and unstable GudB protein in *Bacillus subtilis*

**DOI:** 10.3389/fmicb.2014.00758

**Published:** 2015-01-07

**Authors:** Lorena Stannek, Katrin Gunka, Rachel A. Care, Ulf Gerth, Fabian M. Commichau

**Affiliations:** ^1^Department of General Microbiology, Georg-August-University GöttingenGöttingen, Germany; ^2^Institute of Microbiology, Ernst-Moritz-Arndt-University GreifswaldGreifswald, Germany

**Keywords:** proteolysis, arginine phosphorylation, protein modification, protein folding, glutamate dehydrogenase

## Abstract

The Gram-positive model bacterium *Bacillus subtilis* contains two glutamate dehydro genase-encoding genes, *rocG* and *gudB*. While the *rocG* gene encodes the functional GDH, the *gudB* gene is cryptic (*gudB^CR^*) in the laboratory strain 168 due to a perfect 18 bp-long direct repeat that renders the GudB enzyme inactive and unstable. Although constitutively expressed the GudB^CR^ protein can hardly be detected in *B. subtilis* as it is rapidly degraded within stationary growth phase. Its high instability qualifies GudB^CR^ as a model substrate for studying protein turnover in *B. subtilis*. Recently, we have developed a visual screen to monitor the GudB^CR^ stability in the cell using a GFP-GudB^CR^ fusion. Using fluorescent microscopy we found that the GFP protein is simultaneously degraded together with GudB^CR^. This allows us to analyze the stability of GudB^CR^ in living cells. By combining the visual screen with a transposon mutagenesis approach we looked for mutants that show an increased fluorescence signal compared to the wild type indicating a stabilized GFP-GudB^CR^ fusion. We observed, that disruption of the arginine kinase encoding gene *mcsB* upon transposon insertion leads to increased amounts of the GFP-GudB^CR^ fusion in this mutant. Deletion of the cognate arginine phosphatase YwlE in contrast results in reduced levels of the GFP-GudB^CR^ fusion. Recently, it was shown that the kinase McsB is involved in phosphorylation of GudB^CR^ on arginine residues. Here we show that selected arginine-lysine point mutations of GudB^CR^ exhibit no influence on degradation. The activity of McsB and YwlE, however, are crucial for the activation and inhibition, respectively, of a proteolytic machinery that efficiently degrades the unstable GudB^CR^ protein in *B. subtilis*.

## INTRODUCTION

Posttranslational modifications of proteins allow bacteria to control several important cellular processes. Phosphorylation is such a posttranslational modification event that can severely affect the function of a protein, which is targeted by a specific kinase (Pawson and Scott, 2005; Jers et al., 2008; Kobir et al., 2011). In bacteria, phosphorylation of enzymes and of enzyme regulators is important for the re-direction of fluxes through central metabolic pathways (LaPorte and Koshland, 1983; Cozzone and El-Mansi, 2005; Niebisch et al., 2006). Moreover, posttranslational modification of RNA- and DNA-binding transcription factors by phosphorylation may result in induction or repression of gene expression (Bird et al., 1993; Stülke et al., 1997; Jung et al., 2012; Mascher, 2014).

In the past years, several studies revealed that beside serine, threonine, histidine, and cysteine also amino acids like tyrosine and arginine are phosphorylated in bacteria (Meins et al., 1993; Hoch, 2000; Deutscher and Saier, 2005; Macek et al., 2007; Kobir et al., 2011). For instance, the activity of the UDP-glucose dehydrogenase in the Gram-positive model bacterium *Bacillus subtilis* is controlled by reversible phosphorylation of a tyrosine residue (Mijakovic et al., 2004). Phosphorylation of tyrosine residues has also been shown to be important for controlling the activity of DNA-binding proteins (Mijakovic et al., 2006; Derouiche et al., 2013). Recently, phosphoproteomic studies revealed that phosphorylation of arginine residues is an emerging posttranslational modification, which is implicated in general stress response in *B. subtilis* (Elsholz et al., 2012; Schmidt et al., 2014; Trentini et al., 2014). The kinase responsible for arginine phosphorylation in *B. subtilis* was shown to be McsB (Fuhrmann et al., 2009). Under normal growth conditions McsB is bound and inhibited by the ClpC ATPase subunit of the ClpCP protease complex and/or the activator of McsB kinase activity, McsA. At the same time, the DNA-binding transcription factor CtsR represses the genes of the CtsR-regulon (Derré et al., 1999). In contrast, if the bacteria encounter heat stress, ClpC preferentially interacts with misfolded proteins and releases McsB, which finally targets CtsR for degradation (Kirstein et al., 2005). Inactivation of CtsR results in upregulation of genes that encode proteins of a central protein quality network. The proteins of this network include chaperones, proteases, and adaptor proteins that improve the recognition of substrates by proteases (Elsholz et al., 2010a; Battesti and Gottesmann, 2013). Recent findings indicate that the detachment of CtsR from the DNA provoked by heat seems to be mediated by an intrinsic protein domain that senses heat rather than by McsB-dependent phosphorylation of arginine residues (Elsholz et al., 2010b). By contrast, upon oxidative stress, McsA does not longer bind to and inhibit McsB, which subsequently removes CtsR from the DNA (Elsholz et al., 2011). Thus, the way of how the DNA-binding activity of CtsR is controlled by oxidative stress and by heat is strikingly different.

In recent global phosphoproteomic studies using a *B. subtilis ywlE* mutant strain lacking the cognate phosphatase YwlE of the kinase McsB, several arginine phosphorylation sites were detected (Elsholz et al., 2012; Schmidt et al., 2014; Trentini et al., 2014). Two phosphorylatable arginine residues in the ClpC protein were shown to be important for McsB-dependent activation of the ATPase subunit of the ClpCP protease complex (Elsholz et al., 2012). In the same study it has been shown that the arginine kinase McsB and the cognate phosphatase YwlE may influence the expression of different global regulons. However, the impact of arginine phosphorylation on the physiology of *B. subtilis* is not yet fully understood. Analyses of the dynamic changes in the arginine phosphoproteome in response to heat and oxidative stress revealed that only a minor fraction of the phosphorylation sites were differentially modified (Schmidt et al., 2014).

We are interested in the regulation of glutamate metabolism in *B. subtilis*. In addition to *de novo* synthesis of the important amino group donor glutamate, the bacteria may use glutamate as a source of carbon and nitrogen (for a recent review see Gunka and Commichau, 2012). Utilization of glutamate requires expression of the *rocG* and *gudB* genes encoding the catabolically active glutamate dehydrogenases (GDHs) RocG and GudB, respectively (Belitsky and Sonenshein, 1998; Gunka et al., 2013). Some isolates of *B. subtilis* like the “wild” ancestor strain NCIB3610 indeed synthesize two active GDHs allowing the bacteria to use glutamate as the single source of carbon and nitrogen (Zeigler et al., 2008; unpublished results). In the domesticated *B. subtilis* strain 168 only the *rocG* gene encodes a functional GDH (Belitsky and Sonenshein, 1998; Zeigler et al., 2008). In this strain, the *gudB^CR^* gene is cryptic (CR) due to a perfect 18 bp-long direct repeat (DR). This occurs in the part of the gene encoding the active center of the enzyme (Belitsky and Sonenshein, 1998). The GudB^CR^ is enzymatically inactive and also subject to rapid proteolytic degradation, especially when the bacteria starve for nutrients, which is the case when bacteria enter stationary phase (Gerth et al., 2008; Gunka et al., 2012, 2013). Although ClpP was shown to slightly affect GudB^CR^ stability (Gerth et al., 2008), other factors that are involved in the recognition and degradation of the protein are unknown. Interestingly, McsB was shown to phosphorylate the inactive GudB^CR^ protein on four arginine residues (Elsholz et al., 2012). It is tempting to speculate that this phosphorylation serves as a label that directs the inactive GudB^CR^ protein to the proteolytic machinery (see below).

In the present study, we apply a visual screen that is based on a GFP-GudB^CR^ fusion to monitor the GudB^CR^ stability *in vivo*. By applying microscopical and biochemical techniques, we found that GFP and GudB^CR^ are simultaneously degraded. Thus, the visual screen is suitable to analyze the cellular amount of GudB^CR^. To identify novel factors that are involved in GudB^CR^ degradation, we combined the visual screen with a transposon mutagenesis approach. Afterward we looked for mutants that show an increased fluorescence, indicating increased amounts of the GFP-GudB^CR^ fusion. Among the transposants we found one insertion in the *mcsB* gene encoding the arginine kinase McsB. Moreover, inactivation of the cognate phosphatase YwlE resulted in a decreased fluorescence of a strain synthesizing the GFP-GudB^CR^ fusion. The possible mechanisms of how the activity of the kinase McsB and the cognate phosphatase YwlE affect the amount of the GudB^CR^ protein are discussed.

## MATERIALS AND METHODS

### CHEMICALS, MEDIA, AND DNA MANIPULATION

The oligonucleotides were purchased from Sigma-Aldrich (Germany) and are listed in **Table 1**. *B. subtilis* chromosomal DNA was isolated using the DNeasy Blood & Tissue Kit (Qiagen, Germany). Plasmids were isolated from *Escherichia coli* using the Nucleospin Extract Kit (Macherey and Nagel, Germany). PCR products were purified using the PCR Purification Kit (Qiagen, Germany). Phusion DNA polymerase, restriction enzymes and T4 DNA ligase were purchased from Thermo Scientific (Germany) and used according to the manufacturer’s instructions. Other chemicals and media were purchased from Sigma-Aldrich (Germany), Carl Roth (Karlsruhe, Germany) and Becton Dickinson (Heidelberg, Germany). Sequencing of DNA was performed by the SeqLab Sequence Laboratories (Göttingen, Germany).

**Table 1:** Oligonucleotides used in this study.

Name	Sequence	Purpose
KG166	5′-GCGGGATACGTTTTCACC	direct repeat analysis
KG167	5′-CACCGCCATATGGAAGATC	direct repeat analysis
KG180	5′-TTTGTATAGTTCATCCATGCCATGTGTAATC	Construction of plasmid pBP187
KG181	5′-ACATGGCATGGATGAACTATACAAA ATGGCAGCCGATCGAAACACCG	Construction of plasmid pBP187
KG184	5′-AAAGAATTCTCATTATATCCAGCCTCTAAAACGCG	Construction of plasmid pBP4
KG185	5′-TTTGGATCCCATTCAGCTTTCAGAAAGCTTACAGCGAATC	Construction of plasmid pBP4
KG188	5′-AAACAATTGCATTCAGCTTTCAGAAAGCTTACAGCGAATC	Construction of plasmid pBP184
KG190	5′-AAAGAATTCAAAGGAGGAAACAATCATGAGTAAAGG AGAAG AACTTTTCACT	Construction of plasmid pBP187
LS92	5′-AAAGGATCCAATAGAGAAAAATAAGGGGTGA CTGACATGGATATTA	Construction of plasmid pBP183
LS93	5′-TTTCTGCAGTTATCTACGGTCTTTTTTCAGCTGTTTTGCCAG	Construction of plasmid pBP183
LS94	5′-P-TAACGGTAAAAATACCTGTTAAGATGGACGAC GGTTCAGTAAAG	Construction of plasmid pBP184
LS95	5′-P-AACGAAAGGCGGGATAAAGTTTCACCCGAACGTAACA	Construction of plasmid pBP184
LS96	5′-TTTGGATCCTTATATCCAGCCCTTAAACTTCGAAGCTT CAGCCATTTTG	Construction of plasmid pBP184
LS97	5′-AAAGGATCCGTACAGATAGTGAGGAGGAACAGGAGTAA	Construction of plasmid pBP186
LS98	5′-TTTCTGCAGTCATATCGATTCATCCTCCTGTCTTTTCCC	Construction of plasmid pBP186
pIC333_seq up	5′-AAGAGCGCCCAATACGCAAACCGCC	Sequencing transposon plasmids
pIC333_seq down	5′-TTTGCATGCTTCAAAGCCTGTCGGAATTGG	Sequencing transposon plasmids

### BACTERIAL STRAINS, GROWTH CONDITIONS, AND CONSTRUCTION OF MUTANT STRAINS

*B. subtilis* strains (**Table 2**) were grown in LB and SP medium, respectively. LB and SP plates were prepared by the addition of 17 g agar/l (Roth, Germany) to LB and SP (8 g nutrient broth/l, 1 mM MgSO_4_, 13 mM KCl, supplemented after sterilization with 2.5 μM ammonium ferric citrate, 500 μM CaCl_2_, and 10 μM MnCl_2_), respectively. When required, media were supplemented with antibiotics at the following concentrations: ampicillin (100 μg/ml), kanamycin (10 μg/ml), chloramphenicol (5 μg/ml), lincomycin/erythromycin (25/2 μg/ml), tetracyclin (12.5 μg/ml), and spectinomycin (150 μg/ml). *B. subtilis* was transformed with plasmid and chromosomal DNA according to a previously described two-step protocol (Kunst and Rapoport, 1995).

**Table 2:** *Bacillus subtilis* strains used in this study.

Name	Relevant genotype	Reference or source^a^
168	*trpC2*	Laboratory collection
BP25	*trpC2 Δ gudB::aphA3 amyE::(gfp-gudB^CR^ cat)*	pBP8 → GP1160
BP26	*trpC2 ΔgudB::aphA3 amyE::(gfp-gudB cat)*	pBP9 → GP1160
BP69	*trpC2 ΔgudB::aphA3 mcsB::*Tn*10 spc amyE::(gfp-gudB^CR^ cat)*	pIC333 → BP25
BP74	*trpC2 ΔgudB::aphA3 ywlE::tet amyE::(gfp-gudB^CR^ cat)*	GP1459 → BP25
BP75	*trpC2 ΔgudB::aphA3 ywlE::tet amyE::(gfp-gudB cat)*	GP1459 → BP26
BP98	*trpC2 ΔgudB::aphA3 amyE::(gfp-gudB cat) clpC::spc*	*clpC::spc* → BP25
BP99	*trpC2 ΔgudB::aphA3 amyE::(gfp-gudB cat) clpP::tet*	*clpP::tet* → BP25
BP230	*trpC2 ΔgudB::aphA3 amyE::(gfp-gudB^CR^* R56K, R83K, R421K, R423K *cat)*	pBP187 → GP1160
BP231	*trpC2 ΔgudB::aphA3 mcsB::*Tn*10 spc amyE::(gfp-gudB^CR^* R56K, R83K, R421K, R423K *cat)*	BP69 → BP230
BP311	*trpC2 ΔgudB::aphA3 mcsB::*Tn*10 spc amyE::(gfp-gudB cat)*	pBP45 → BP26
GP1160	*trpC2 ΔgudB::aphA3*	Gunka et al. (2012)
GP1459	*trpC2 ΔywlE*::*tet*	BDO01 → 168

^a^Arrows indicate construction by transformation.

### CONSTRUCTION OF PLASMIDS

The plasmids for complementation of the *ywlE* and *mcsB* mutations in *B. subtilis* were constructed as follows. The *ywlE* and *mcsB* genes were amplified by PCR from chromosomal DNA using the oligonucleotide pairs LS92/LS93 and LS97/LS98, respectively (**Table 1**). The PCR products were digested with the enzymes *Bam*HI and *Pst*I and ligated to the plasmid pBQ200 that was cut with the same enzymes. The plasmids harboring the *ywlE* and *mcsB* genes and their native ribosome-binding sites were designated pBP183 and pBP186, respectively. Expression of the genes is driven by the constitutively active *P_degQ_* promoter (Martin-Verstraete et al., 1994). The quadruple *gfp-gudB^CR^* mutant (designated as *gfp-gudB^CR^*-mut), encoding the GudB^CR^ protein in which the arginine residues 56, 83, 421, and 423 were replaced by lysine, was constructed by the Multiple-mutation reaction (MMR; Hames et al., 2005). The mutated *gudB^CR^* allele was amplified with the oligonucleotide pair KG188/LS96 and the mutagenic oligonucleotides LS94, LS95, and LS96 using plasmid pBP4 as a template. The MMR product was digested with the enzymes *Mfe*I and *Bam*HI, and ligated to the plasmid pAC5 that was cut with the enzymes *Eco*RI and *Bam*HI. The resulting plasmid was designated as pBP184. This plasmid was used to amplify the promoterless quadruple *gudB^CR^* mutant allele by PCR using the oligonucleotide pair KG181/LS96. The *gfp* gene containing the ribosome-binding site of the *B. subtilis gapA* gene was amplified by PCR from plasmid pBP8 using the oligonucleotide pair KG180/KG190. The *gfp* and *gudB^CR^* genes were fused by PCR using the external oligonucleotides KG190 and LS96, the PCR product was digested with *Bam*HI and *Eco*RI, and ligated to the plasmid pBP7 that was cut with the same enzymes. The resulting plasmid pBP187 contains the native *gudB* promoter and integrates in single copy into the *amyE* locus. Replacement of the arginine codons in the *gfp-gudB^CR^* gene was confirmed by DNA sequencing. All cloning procedures were performed with the *E. coli* strain DH5α (Sambrook et al., 1989).

### TRANSPOSON MUTAGENESIS

For transposon mutagenesis of the *B. subtilis* strain BP25, we used the mini-Tn*10* delivery vector pIC333 (Steinmetz and Richter, 1994) as described previously (Chauvaux et al., 1998). The transposants were grown on SP agar plates for 48 h at 42°C and the intensity of the GFP signal was evaluated by stereo fluorescence microscopy. For the determination of the site of mini-Tn*10* insertion, we made use of the fact that the integrated DNA fragment does not contain any *Eco*RI restriction sites. The chromosomal DNA of the mutants was digested with *Eco*RI and re-ligated. The ligation mixture was used to transform *E. coli* DH5α (Sambrook et al., 1989). For all mutants that were further analyzed, we obtained plasmids conferring spectinomycin resistance (**Table 3**). The insertion sites of the mini-Tn*10* transposon were determined by DNA sequencing of the plasmids using the oligonucleotides pIC333_seq up and pIC333_seq down.

**Table 3:** Plasmids used and constructed in this study.

Name	Purpose	Reference or source
pIC333	Transposon mutagenesis	Steinmetz and Richter (1994)
pAC5	Integration of DNA into the *amyE* locus	Martin-Verstraete et al. (1992)
pBQ200	Complementation studies	Martin-Verstraete et al. (1994)
pBP4	*PgudB^CR^ -gudB^CR^* in pAC5	This work
pBP7	*PgudB^CR^* in pAC5	Gunka et al. (2013)
pBP8	*gfp-gudB^CR^* in pBP7	Gunka et al. (2013)
pBP45	Transposon plasmid *mscB*	This work
pBP183	Expression of *ywlE*	This work
pBP184	Expression of *gudB^CR^* (R56K R83K R421K R423K)	This work
pBP186	Expression of *mcsB*	This work
pBP187	Expression of *gfp-gudB^CR^*(R56K R83K R421K R423K)	This work

### WESTERN BLOTTING

For Western blot analyses, proteins present in 20–50 μg of cell free crude extracts were separated by 12.5% SDS PAGE and transferred onto polyvinylidene difluoride membrane (BioRad, Germany) by semi-dry electroblotting. Anti-GFP (PromoKine, Germany; MBL, Japan), anti-YwlE, anti-McsB, and anti-GapA polyclonal antibodies were diluted 1:10.000, 1:1000, 1:5.000, and 1:30.000, respectively, and served as primary antibodies. The antibodies were visualized using anti-rabbit immunoglobulin alkaline phosphatase secondary antibodies (Promega, Germany) and the CDP-Star detection system (Roche Diagnostics, Switzerland) as described previously (Commichau et al., 2007).

### FLUORESCENCE MICROSCOPY

For fluorescence microscopy, cells were grown in SP medium to optical densities as indicated, and analyzed on agarose microscopy slides. Fluorescence images were obtained with an Axioskop 40 FL fluorescence microscope, equipped with digital camera AxioCam MRm and AxioVision Rel (version 4.8) software for image processing (Carl Zeiss, Göttingen, Germany) and Neofluar series objective at x 100 primary magnification. The applied filter set was eGFP HC-Filterset (band-pass [BP] 472/30, FT 495, and long-pass [LP] 520/35; AHF Analysentechnik, Tübingen, Germany) for GFP detection. Pictures of *B. subtilis* colonies were taken with a stereo fluorescence microscope Lumar.V12 (Zeiss, Jena, Germany) equipped with the ZEN lite 2011 (blue edition) software. The applied filter set was Lumar 38 for eGFP detection (Zeiss, Jena, Germany). Images were taken at room temperature and an exposure time of 1 s.

### MONITORING GFP-GudB^CR^ LEVELS IN GROWING CULTURES

Cellular amounts of the GFP-GudB^CR^ fusion protein were determined by monitoring the fluorescence (excitation 489/9.0 nm, emission 509/9.0 nm) in a growing bacterial culture using the Synergy MX II multimode microplate reader (BioTek). For this purpose, 4 ml LB medium were inoculated with the precultures to an OD_600_ of 0.1. The cultures, that had an approximate OD_600_ of 1.0, were used to inoculate a 96 well plate (Corning, Sigma) containing 180 μl medium per well. To avoid evaporation, the outermost wells were filled with 200 μl sterile water. The plates were incubated for a maximum of 10 h at 37°C and fast shaking speed. OD_600_ was measured every 10 min throughout the experiment. Background fluorescence of the parental strains was subtracted from the raw fluorescence of all *gfp* fusion strains at the same OD_600_. The cellular amounts of the GFP-GudB^CR^ fusion protein correspond to the fluorescence divided by the OD_600_ at each time point.

## RESULTS

### A STABLE SCREENING SYSTEM FOR IDENTIFYING FACTORS INVOLVED IN GudB^CR^ DEGRADATION

**FIGURE 1:**
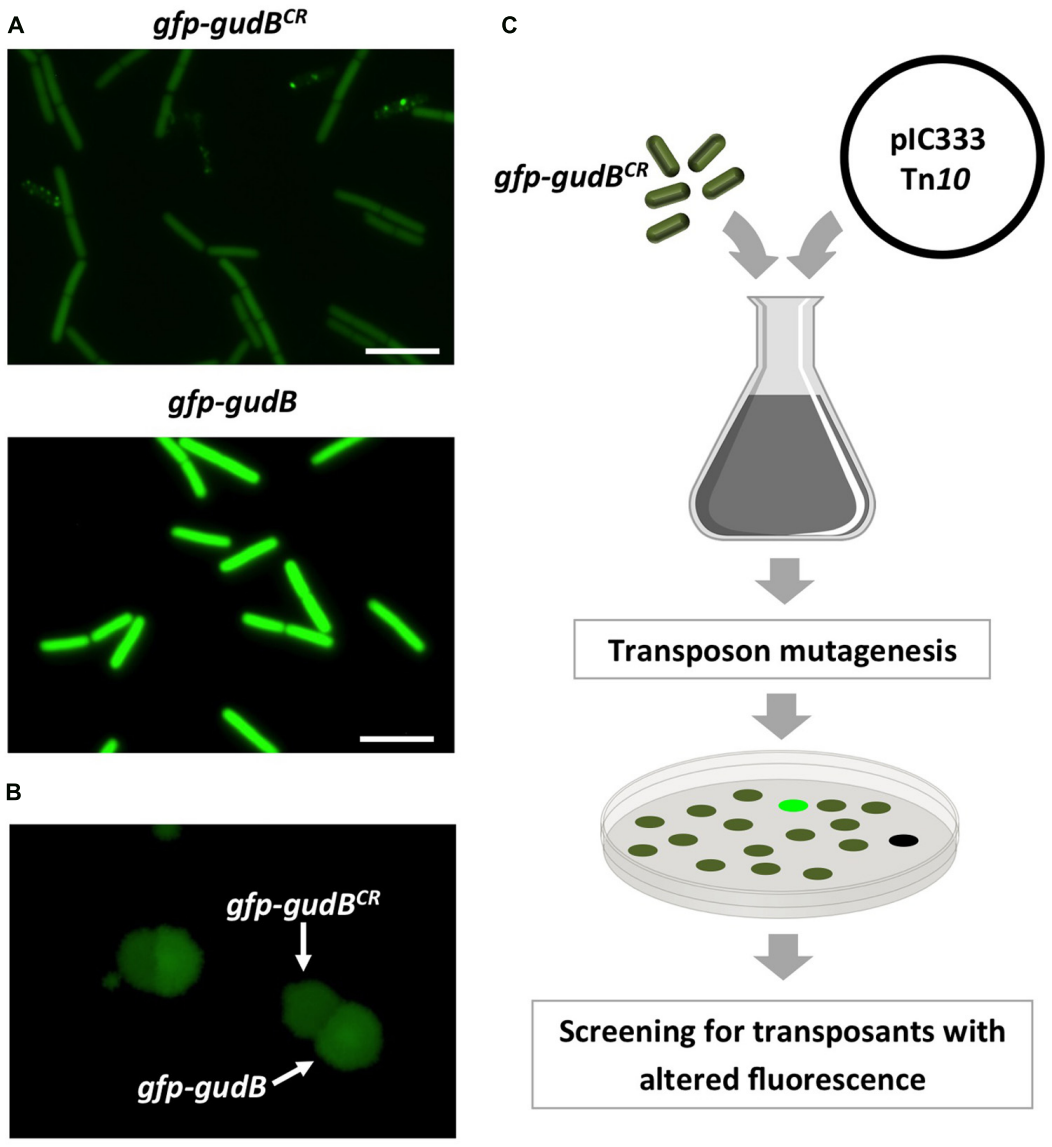
**Fluorescence of strains BP25 (*gfp-gudB^**CR**^*) and BP26 (*gfp-gudB*) synthesizing the GFP-GudB^**CR**^ and GFP-GudB proteins, respectively, at the single cell **(A)** and at the colony level **(B)**; transposon mutagenesis to identify factors involved in GFP-GudB^**CR**^ degradation **(C)**.** For single cell analysis the bacteria were grown in SP medium. Exposure time, 5 s; scale bar, 5 μm. To monitor fluorescence of colonies the strains were grown in SP medium, mixed and appropriate dilutions were propagated on SP plates, which were incubated for 24 h at 37°C. Exposure time, 1 s.

The fact that also the GFP-GudB^CR^ protein is degraded (Gunka et al., 2012, 2013) qualifies it as a substrate to uncover the proteolytic machinery. Before identifying factors that contribute to GudB^CR^ degradation, we constructed the *rocG* plus strain BP25 that is genetically stable (Gunka et al., 2012) and synthesizes the active GDH RocG as well as the inactive GFP-GudB^CR^ fusion. To test if the GFP-GudB^CR^ fusion protein is degraded in this strain, we compared the fluorescence signal of cells to those of strain BP26 harboring the active *gfp-gudB* fusion. As shown in **Figure 1A**, while the bacteria with the active GFP-GudB fusion were strongly fluorescent, the fluorescence signal of bacteria synthesizing the inactive GFP-GudB^CR^ protein was reduced. Thus, the inactive GFP-GudB^CR^ fusion is also degraded in the new strain background. We also tested whether the two strains can be distinguished from each other by monitoring the fluorescence emitted by colonies that were grown on rich medium agar plates. For this purpose, the strains BP25 (*gfp-gudB^CR^*) and BP26 (*gfp-gudB*) were grown in liquid medium, mixed in a 1:1 ratio and appropriate dilutions were propagated on SP plates to allow growth of individual colonies. By visual inspection of the plates using a stereo fluorescence microscope we found several colonies that were grown close to each other and showed different fluorescence signals (**Figure 1B**). We then re-streaked some of the colonies showing different fluorescence signals on agar plates to obtain individual colonies. Next, we performed colony PCRs and confirmed that the higher and lower fluorescence signals were due to the presence of the *gfp-gudB* and *gfp-gudB^CR^* alleles, respectively. In conclusion, the visual screen seems to be suitable to look for mutants, lacking factors that enhance or decrease proteolytic degradation of GFP-GudB^CR^.

### IDENTIFICATION OF MscB CONTRIBUTING TO GudB^CR^ DEGRADATION

**FIGURE 2:**
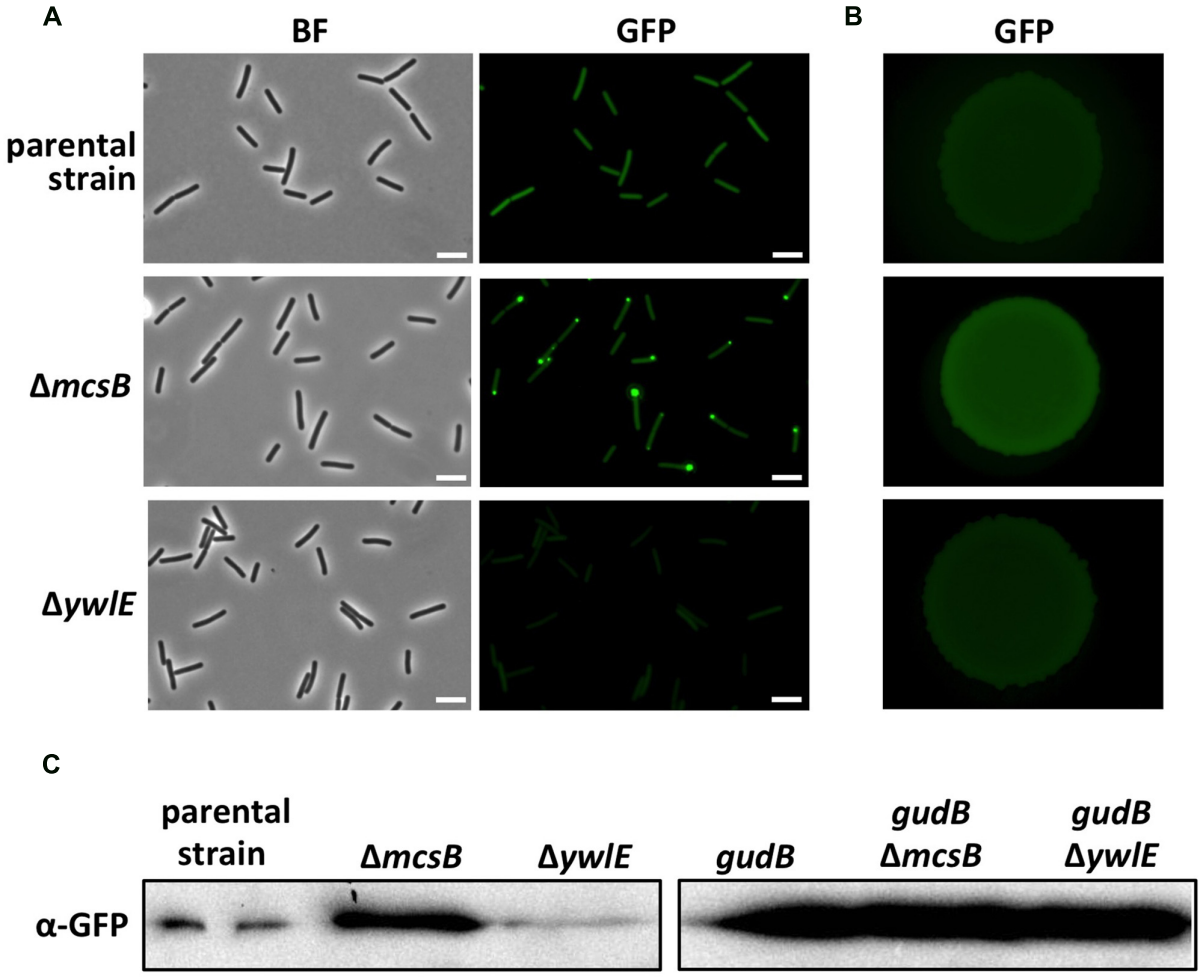
**Evaluation of the GFP-GudB^**CR**^/GFP-GudB levels by fluorescence microscopy and Western blotting.** For fluorescence microscopic analyses of single cells **(A)** the parental strain B25 (*gfp-gudB^CR^*) and the strains BP69 (*gfp-gudB^CR^ mcsB*) and BP74 (*gfp-gudB^CR^ ywlE*) were grown in SP medium. For bright field and fluorescence microscopy the exposure times were 150 ms and 5 s, respectively. For the evaluation of the GFP-GudB^CR^ level by stereo fluorescence microscopy **(B)** the bacteria were grown in SP medium until stationary growth phase and 10 μl of cell suspensions with an approximate OD_600_ of 1 were dropped on a SP plate. The plate was incubated for 24 h at 37°C. Exposure time, 1 s; scale bar, 5 μm. For Western blot analysis **(C)** the strains B25 (*gfp-gudB^CR^*), BP69 (*gfp-gudB^CR^ mcsB*) and BP74 (*gfp-gudB^CR^ ywlE*) as well as the isogenic strains BP26 (*gfp-gudB*), BP311 (*gfp-gudB mcsB*) and BP75 (*gfp-gudB ywlE*) expressing the active *gfp-gudB* fusion were grown in SP medium and 30 μg of the cell free crude extracts were loaded onto a 12.5% SDS PAGE. The fusion proteins were detected using GFP polyclonal antibodies.

To identify factors that are involved in degradation or stabilization of GudB^CR^, we performed a transposon mutagenesis with strain BP25 (*gfp-gudB^CR^*) using the mini-Tn*10* delivery vector pIC333 (Steinmetz and Richter, 1994). Afterward, we screened for mutants that show an altered fluorescence signal using a stereo fluorescence microscope (**Figure 1C**). Appropriate dilutions of the transposants were propagated on SP plates that were incubated for 48 h at 42°C. By visual inspection of about 8000 transposants we could identify one mutant that showed no fluorescence signal, whereas a second mutant showed an increase in fluorescence intensity. While the first mutant had obviously lost the ability to synthesize GFP because the transposon was inserted into the *gfp* gene, the mutant showing increased fluorescence had integrated the transposon at position 580 into the arginine kinase encoding *mcsB* gene (Fuhrmann et al., 2009). This transposon mutant was designated as BP69. A re-evaluation of the fluorescence signal of single cells and of a colony of the *mcsB* transposon mutant revealed that the cellular amount of the GFP-GudB^CR^ fusion was increased when compared to that of the parent strain BP25 (**Figures 2A,B**). The lack of McsB also resulted in the formation of large aggregates of the GFP-GudB^CR^ fusion protein at the cell poles (**Figure 2A**), an observation that can be made when aggregation prone proteins are synthesized in bacteria (Rokney et al., 2009; Villar-Pique et al., 2012). In conclusion, using transposon mutagenesis in combination with a visual screen, we identified the arginine kinase McsB being a novel factor that contributes to GudB^CR^ degradation.

### McsB AND YwlE ARE INVOLVED IN GudB^CR^ STABILITY

**FIGURE 3:**
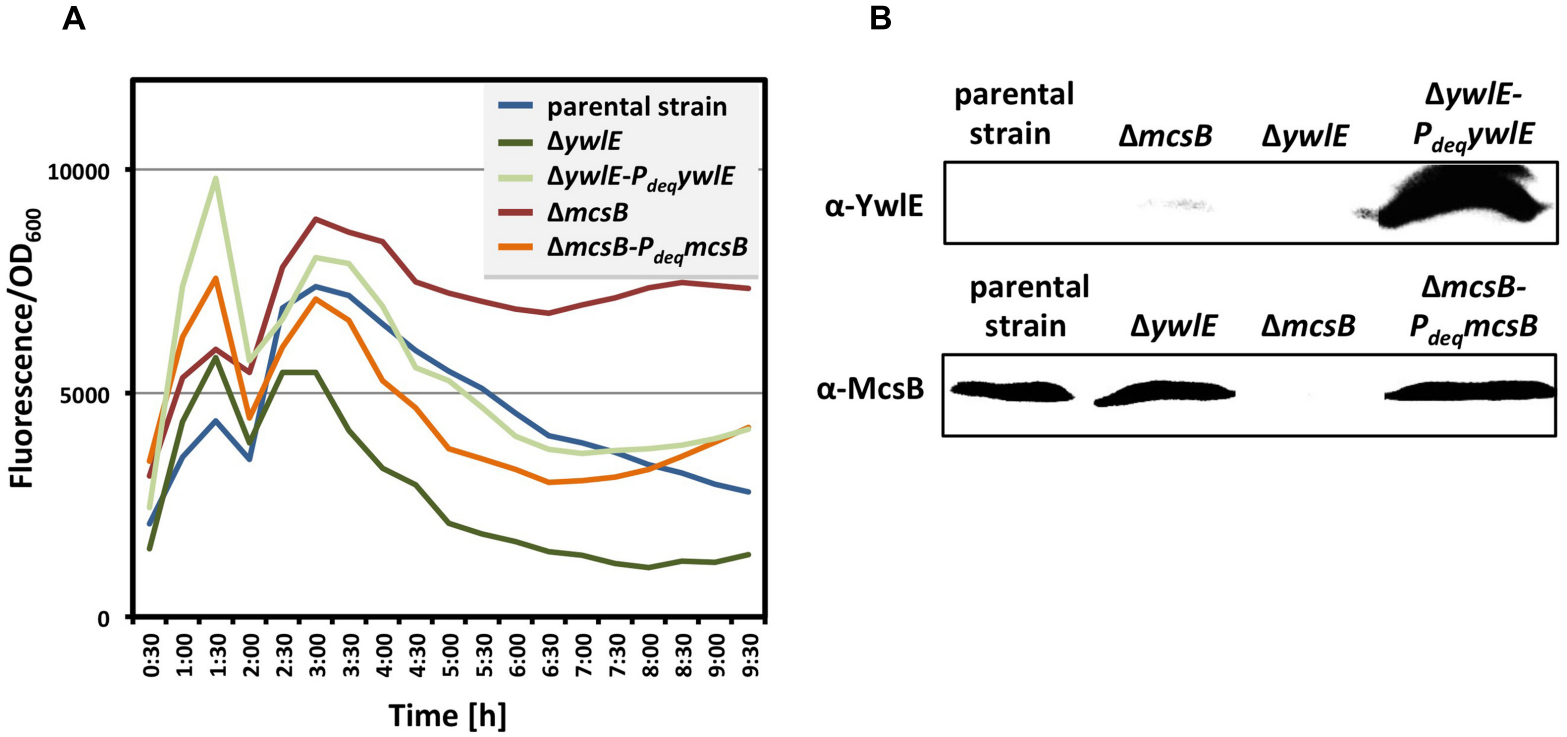
**Complementation of the *mcsB* and *ywlE* mutations.** To verify the complementation of the *mcsB* and the *ywlE* mutations *in vivo* **(A)**, the strains BP69 (*gfp-gudB^CR^ mcsB*) and BP74 (*gfp-gudB^CR^ ywlE*), and the isogenic strains BP69-pBP186 and BP74-pBP183 expressing *mcsB* and *ywlE* from the overexpression vector pBP200 were grown in SP medium and the relative cellular levels of the GFP-GudB^CR^ fusion is reflected by the GFP signal divided by the OD_600_. The parental strain BP25 (*gfp-gudB^CR^*) served as a control. All strains entered stationary phase around 6 h of growth. The maximum deviation of the series of representative data shown here was <30%. For the Western blot analysis **(B)** the bacteria were cultivated in SP medium. 40 μg and 50 μg of the cell free crude extracts were loaded onto a 12.5% SDS PAGE for the detection of the YwlE and McsB proteins, respectively, using polyclonal antibodies.

To underpin the role of arginine phosphorylation in the degradation of the GudB^CR^ protein, we inactivated the *ywlE* gene in the strain BP25 (*gfp-gudB^CR^*). In case the arginine phosphatase YwlE counteracts the function of its cognate kinase McsB, we expected to observe that single cells as well as colonies of the *ywlE* mutant BP74 would show a reduced fluorescence. This was indeed the case for single cells of the *ywlE* mutant strain in comparison to cells of the *mcsB* mutant and parent strains BP69 and BP25, respectively (**Figure 2A**). Although less pronounced, fluorescence of the *ywlE* mutant was also reduced at the level of single colonies (**Figure 2B**). However, a quantification of the fluorescence of the GFP-GudB^CR^ fusion protein monitored in growing cultures in the *ywlE* mutant strain clearly demonstrates that the phosphatase YwlE affects GudB^CR^ stability (see below, **Figure 3A**).

Next, we confirmed that the kinase McsB and the phosphatase YwlE affect the cellular levels of the GFP-GudB^CR^ fusion protein. For this purpose, we cultivated the parent strain BP25 as well as the *mcsB* and *ywlE* mutant strains BP69 and BP74, respectively, in SP medium until stationary phase (the OD_600_ was around 3.0) and analyzed the amounts of the GFP-GudB^CR^ fusion protein by Western blotting using GFP-specific antibodies. As shown in **Figure 2C**, in strain BP69 lacking McsB the cellular amount of the GFP-GudB^CR^ fusion protein was strongly increased. By contrast, the inactivation of the *ywlE* gene resulted in a decrease of GFP-GudB^CR^ levels. In conclusion, the semi-quantitative Western Blot analyses are in perfect agreement with the fluorescence microscopical studies.

### McsB AND YwlE DO NOT INFLUENCE THE CELLULAR LEVELS OF THE ACTIVE GudB PROTEIN

Subsequently, we wanted to answer the question of whether McsB and YwlE do also influence the cellular amounts of the enzymatically active GFP-GudB fusion protein lacking the duplication of three amino acids in the active center of the enzyme. For this purpose, we cultivated the parent strain BP26 (*gfp-gudB*) synthesizing the active GFP-GudB fusion and the isogenic *mcsB* and *ywlE* mutant strains BP311 and BP75 (**Table 2**), respectively, in SP medium until stationary phase (OD_600_ of about 3.0). Afterward, we quantified the amount of the GFP-GudB protein by Western blotting using antibodies specific for GFP. As shown in **Figure 2C**, irrespective of the absence of either McsB or YwlE all strains synthesized similar amounts of the active GFP-GudB fusion protein. In conclusion, only the cellular amount of the inactive GFP-GudB^CR^ but not that of the active GFP-GudB fusion protein is significantly affected by McsB.

### COMPLEMENTATION OF THE *mcsB* and *ywlE* MUTATIONS

For complementation studies of the *mcsB* and *ywlE* mutant strains BP69 and BP74, respectively, we constructed the plasmids pBP186 (*mcsB*) and pBP183 (*ywlE*). Both plasmids are derivatives of the non-integrative overexpression plasmid pBQ200 and gene expression is driven by the constitutively active *P_degQ_* promoter (Martin-Verstraete et al., 1994). The plasmids pBP186 and pBP183 were introduced into the corresponding mutant strains by transformation. Next, we compared the cellular amounts of the GFP-GudB^CR^ fusion protein in the *mcsB* and *ywlE* mutant strains BP69 and BP74, respectively, with those of the isogenic complementation strains by monitoring the fluorescence, which reflects the cellular amounts of the GFP-GudB^CR^ fusion protein during growth of the bacteria. The parent strain BP25 (*gfp-gudB^CR^*) served as a control. As shown in **Figure 3A**, the emitted fluorescence of all cultures was similar during exponentially growth. In the stationary phase the fluorescence signal was much higher in the *mcsB* mutant strain BP69 when compared to that of the parent strain BP25. By contrast, inactivation of the *ywlE* resulted in a strong decrease of the fluorescence signal. Overexpression of the *mcsB* and *ywlE* genes in the *mcsB* and *ywlE* mutant strains BP69 and BP74, respectively, restored the fluorescence signal in the stationary phase almost to the extent of the parent strain. Western blot experiments using antibodies specific for McsB and YwlE confirmed overexpression of the arginine kinase and the phosphatase from the complementation plasmids in the *mcsB* and *ywlE* mutant strains BP69 and BP74, respectively (**Figure 3B**). In conclusion, the cultivation experiments to detect the cellular levels of the GFP-GudB^CR^ fusion protein are in good agreement with the previous experiments showing that the lack of the McsB and YwlE results in elevated and reduced levels, respectively, of the inactive GDH. Moreover, together with the Western blot experiments the cultivation experiments also revealed that the *mcsB* and *ywlE* mutations can be complemented by expressing the *mcsB* and *ywlE* genes from plasmids.

### McsB SEEMS TO ACT INDEPENDENTLY OF ClpC and ClpP ON GudB^CR^ DEGRADATION

**FIGURE 4:**
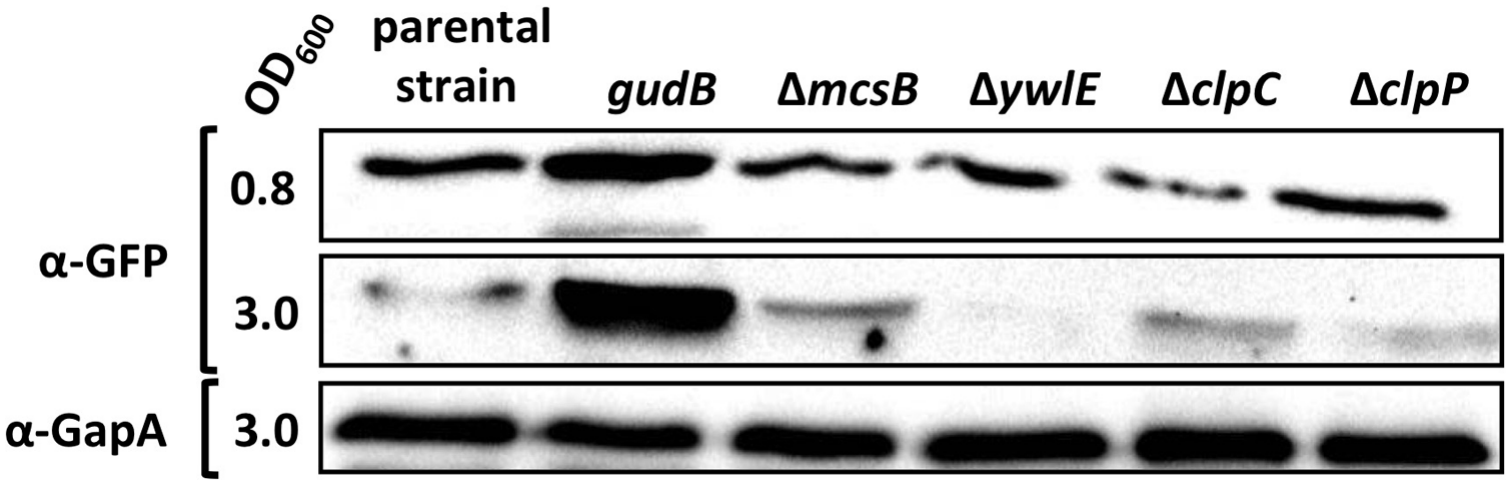
**McsB acts independently of ClpC and ClpP.** For the Western blot analysis the parental strain BP25 (*gfp-gudB^CR^*) and the strains BP26 (*gfp-gudB*), BP69 (*gfp-gudB^CR^ mcsB*), BP74 (*gfp-gudB^CR^ ywlE*), BP98 (*gfp-gudB^CR^ clpC*), and BP99 (*gfp-gudB^CR^ clpP*) were cultivated in SP medium until the indicated optical densities (OD_600_). 30 μg of the cell free crude extracts were loaded onto a 12.5% SDS PAGE for the detection of the GFP and GapA proteins using polyclonal antibodies.

The *mcsB* gene lies immediately upstream of the *clpC* gene in the *ctsR mcsA mcsB clpC* operon. Since the *mcsB* mutation can be complemented, it can be ruled out that enhanced cellular levels of the GFP-GudB^CR^ fusion are a consequence of a polar effect of the transposon insertion into the *mcsB* gene leading to a reduced *clpC* expression and a lower proteolytic activity. However, the lower proteolytic activity in the *mcsB* mutant strain might be due to the missing of McsB-dependent activation of the ClpC-ClpP protease complex. To exclude this possibility, we compared the cellular amounts of the GFP-GudB^CR^ fusion in the background of the *clpC* and *clpP* mutant strains BP98 and BP99, respectively, to that of the parent strain BP25 (*gfp-gudB^CR^*). For this purpose, we grew the bacteria in SP medium and collected samples from exponential and stationary phases and performed Western blot analyses (**Figure 4**). The strain BP26 (*gfp-gudB*) as well as the *mcsB* and *ywlE* mutant strains BP69 and BP74, respectively, served as controls. As expected, in contrast to the inactive GFP-GudB^CR^ fusion protein the active GFP-GudB variant was more abundant during exponential and stationary phase. Moreover, as observed in the previous experiments, the GFP-GudB^CR^ levels were increased and decreased in the *mcsB* and *ywlE* mutants, respectively (see also **Figure 2C**). Finally, the GFP-GudB^CR^ levels in the *clpC* and *clpP* mutant strains BP98 and BP99, respectively, were similar to that of the parent strain BP25 (*gfp-gudB^CR^*). Using GapA and GFP antibodies, we show that only the GFP-GudB^CR^ fusion but not GapA was degraded in stationary growth phase samples. Thus, McsB is involved in GudB^CR^ degradation in a rather ClpP and ClpC-independent manner.

### REPLACEMENT OF PHOSPHORYLATION SITES DOES NOT AFFECT McsB-DEPENDENT GudB^CR^ DEGRADATION

**FIGURE 5:**
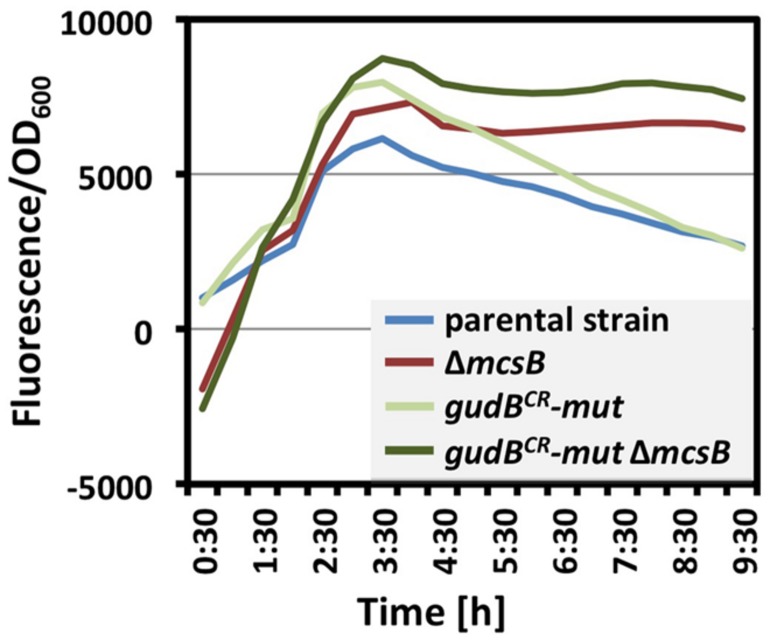
**Impact of McsB on the cellular levels of the GFP-GudB^**CR**^ and GFP-GudB^**CR**^(R56K, R83K, R421K, R423K) proteins.** The strains BP25 (*gfp-gudB^CR^*), BP69 (*gfp-gudB^CR^ mcsB*), BP230 (*gfp-gudB^CR^-mut*), and BP231 (*gfp-gudB^CR^-mut mcsB*) were cultivated in SP medium and the relative cellular levels of the GFP-GudB^CR^ fusion is reflected by the GFP signal divided by the OD_600_. The parental strain BP25 (*gfp-gudB^CR^*) served as a control. All strains entered stationary phase around 6 h of growth. The maximum deviation of the series of representative data shown here was <30%.

In a recent phosphoproteome analysis it has been shown that the inactive GudB^CR^ protein is phosphorylated on the arginine residues at positions 56, 83, 421, and 423 (Elsholz et al., 2012). To evaluate whether phosphorylation of these sites is important for the degradation of the GFP-GudB^CR^ protein, we replaced the arginine by the structurally similar amino acid lysine and monitored the amount of the GudB^CR^ variant *in vivo*. For this purpose the parent strain BP25 (*gfp-gudB*^*CR*^), the *mcsB* mutant strain BP69 (*mcsBgfp-gudB*^*CR*^), the quadruple GFP-GudB^CR^ mutant strain BP230 (*gfp-gudB*^*CR*^-*mut* (R56K R83K R421K R423K)), and the isogenic *mcsB* mutant strain BP231 (*mcsBgfp-gudB*^*CR*^-*mut* (R56K R83K R421K R423K)) were cultivated in SP medium. Simultaneously, the cellular levels of the fusion proteins were determined by monitoring the fluorescence during bacterial growth. As shown in **Figure 5**, the fluorescence measurements revealed that the cellular levels of the fusion proteins in strains BP25 (*gfp-gudB^CR^*) and BP230 (*gfp-gudB^CR^*-mut (R56K R83K R421K R423K)) was much lower in comparison to those of the isogenic *mcsB* mutant strains BP69 (*mcsB gfp-gudB^CR^*), and BP231 (*mcsB gfp-gudB^CR^*-mut (R56K R83K R421K R423K)). In conclusion, these observations suggest that phosphorylation of the arginine residues 56, 83, 421, and 423 sites is rather not important for the degradation of the inactive GudB^CR^ protein.

## DISCUSSION

In the present study, we found that the inactivation of the *mcsB* arginine kinase gene resulted in stabilization of the inactive GDH GudB^CR^ during stationary growth phase of *B. subtilis*. Thus, beside its role in controlling the degradation of the DNA-binding transcription factor CtsR (Elsholz et al., 2010b) and delocalization of proteins involved in the development of transformability of *B. subtilis* (Hahn et al., 2009), McsB activity also mediates degradation of GudB^CR^. Moreover, we found that the arginine phosphatase YwlE counteracts the function of McsB and prevents degradation of GudB^CR^.

There are several possibilities how McsB and YwlE might stimulate and prevent GudB^CR^ degradation, respectively. As it has been reported previously for the proteolytic degradation of CtsR (Elsholz et al., 2012), McsB-dependent activation of the ATPase subunit ClpC of the ClpCP protease complex by phosphorylation of two specific arginine residues could also be crucial for GudB^CR^ degradation. However, according to our Western blot analysis ClpP and ClpC appear apparently not involved in GudB^CR^ degradation. Recent global phosphoproteomic studies have revealed that in the absence of YwlE several proteins, among them the GudB^CR^ protein are phosphorylated on arginine residues (Elsholz et al., 2012; Schmidt et al., 2014; Trentini et al., 2014). These studies prompted us to address the question of whether the phosphorylation of GudB^CR^ by McsB could serve as a label for proteolysis. However, although the cellular levels of the GudB^CR^ quadruple mutant, in which the arginine residues 56, 83, 421, and 423 were replaced by lysine residues, were slightly increased, the protein was still degraded in a McsB-dependent manner when the bacteria entered stationary phase (see **Figure 5**). Thus, the degradation of GudB^CR^ seems to be rather indirectly influenced by McsB. Finally, an unknown proteolytic machinery that remains to be identified might be responsible for the degradation of the misfolded and inactive GDH GudB^CR^. On one hand the activity of the proteolytic machinery might be controlled by McsB-dependent phosphorylation of an unknown adaptor protein that specifically recognizes GudB^CR^ and directs the protein to the protease for degradation. On the other hand McsB could be important for the activation of one of the AAA+ proteases or other unknown proteases that remain to be identified. One could also envision that McsB acts itself as the adaptor that mediates proteolysis of the GudB^CR^ protein. The interaction between McsB and GudB^CR^ could result in coincidental phosphorylation of the GDH. This could also be the case for the other arginine phosphorylations of the *B. subtilis* proteome (Elsholz et al., 2012).

As described above it is interesting to note that only the domesticated *B. subtilis* strains 160, 166, and 168, of which the latter one is used worldwide in basic research and in industry, harbor the *gudB^CR^* gene that is enzymatically inactive and unstable (Zeigler et al., 2008). It has been suggested that the *gudB^CR^* allele appeared as a consequence of *X*-ray mutagenesis and subsequent adaptation for rapid growth of the bacteria in minimal medium lacking the amino group donor glutamate (Burkholder and Giles, 1947). This hypothesis is supported by the observation that a strain that synthesizes in addition to RocG also the enzymatically active GDH GudB is rapidly outcompeted by the laboratory strain 168 (*rocG gudB^CR^*) when exogenous glutamate is not available (Gunka et al., 2013; Stannek et al., 2014). Obviously, the presence of both, RocG and GudB is disadvantageous for the bacteria because the catabolic GDHs degrade the endogenously produced glutamate, which is needed in anabolism. Thus, under laboratory growth conditions a permanent selective pressure must act on the bacteria, which prevents the accumulation of mutants that have spontaneously mutated the cryptic *gudB^CR^* gene and synthesize in addition to RocG the functional GDH GudB. Moreover, the selective pressure acting on the *B. subtilis* strain 168 might be an explanation for the observation that the cryptic *gudB^CR^* gene is stably inherited since the bacterium has been domesticated. Recently, it has been shown that bacteria rapidly loose genes and reduce their genome sizes when adapted to specialized environments. This might also be observed in the laboratory by experimental evolution of bacterial cell populations (Koskiniemi et al., 2012; Lee and Marx, 2012). Therefore, it is somewhat surprising that *B. subtilis* affords to waste energy by permanently synthesizing an inactive enzyme that is subject to rapid degradation. However, under certain growth conditions it must be advantageous for *B. subtilis* to harbor the cryptic *gudB^CR^* gene that, when activated by spontaneous mutagenesis (Gunka et al., 2012), encodes a functional GDH. Indeed, a derivative of the *B. subtilis* 168 expressing *rocG* and *gudB* can use glutamate as a singly source of carbon and nitrogen (Gunka et al., 2013). Thus, under very specific nutritional conditions bacteria that are endowed with high-level GDH activity have a strong selective growth advantage.

In the future it will be interesting to identify additional factors that are involved in the rapid degradation of the enzymatically inactive GDH GudB^CR^. This goal might be achieved by monitoring the cellular amounts of the GFP-GudB^CR^ fusion protein in a mutant collection that have inactivated all non-essential genes by targeted gene deletion or by a next time saturated transposon mutagenesis. The identification of novel factors that are involved in GFP-GudB^CR^ proteolysis might be facilitated by monitoring growth and fluorescence over time because the fusion protein seems to be preferentially degraded in stationary phase. Moreover, it will be interesting to address the question whether arginine phosphorylation influences the physiological functions of other proteins in *B. subtilis*.

## Conflict of Interest Statement

The authors declare that the research was conducted in the absence of any commercial or financial relationships that could be construed as a potential conflict of interest.
